# Ultrasensitive optical absorption in graphene based on bound states in the continuum

**DOI:** 10.1038/srep08266

**Published:** 2015-02-05

**Authors:** Mingda Zhang, Xiangdong Zhang

**Affiliations:** 1 Department of Physics, Beijing Normal University, Beijing 100875, China; 2 School of Physics and Beijing Key Laboratory of Nanophotonics & Ultrafine Optoelectronic Systems, Beijing Institute of Technology, 100081, Beijing, China

## Abstract

We have designed a sphere-graphene-slab structure so that the electromagnetic wave can be well confined in the graphene due to the formation of a bound state in a continuum (BIC) of radiation modes. Based on such a bound state, we have realized strong optical absorption in the monolayer graphene. Such a strong optical absorption exhibits many advantages. It is ultrasensitive to the wavelength because the Q factor of the absorption peak can be more than 2000. By taking suitable BICs, the selective absorption for S and P waves has not only been realized, but also all-angle absorption for the S and P waves at the same time has been demonstrated. We have also found that ultrasensitive strong absorptions can appear at any wavelength from mid-infrared to far-infrared band. These phenomena are very beneficial to biosensing, perfect filters and waveguides.

In recent years, there has been a great deal of interest in studying the optical properties of graphene due to the abundant potential applications within a wide spectral range from terahertz (THz) to visible frequencies^1^,^2^,^3^,^4^,^5^,^6^,^7^,^8^,^9^,^10^,^11^,^12^,^13^,^14^,^15^,^16^,^17^,^18^,^19^,^20^. As an ultra-thin two-dimensional (2D) carbon material, graphene is widely used in the transparent electrodes and optical display materials^1^,^2^,^3^,^4^, it has also been applied in opto-electronics such as photodetectors, optical modulators, and so on^5^,^6^,^7^,^8^,^9^. The strength of interaction between graphene and electromagnetic (EM) waves plays a central role in these applications. However, a single sheet of homogeneous graphene absorbs only 2.3% of normal incidence light in the visible and near-infrared range. It is similar weak in the mid-infrared and far-infrared range. Thus, various methods to improve the interaction between graphene and EM waves have been proposed. For example, the combination of graphene with conventional plasmonic nanostructures^10^,^11^,^12^,^13^,^14^,^15^,^16^, fabricating periodically patterned graphene or placing it in an optical microcavity^17^,^18^,^19^,^20^.

On the other hand, analogous to the localized electrons with energy larger than their potential barriers, light bound states in the continuum (BIC) have also attracted much attention^21^,^22^,^23^,^24^,^25^,^26^,^27^,^28^,^29^. It has recently been shown that it is in principle possible to ideally confine an optical mode within the radiation continuum, realizing an ideal optical bound state surrounded by symmetry-compatible radiation modes^21^,^22^,^23^,^24^,^25^,^26^,^27^,^28^,^29^. Motivated by these investigations, in this work we combine the above two ways, and explore the possibility to realize strong absorption of graphene by using BICs.

## Results and discussion

**Figure 1:**
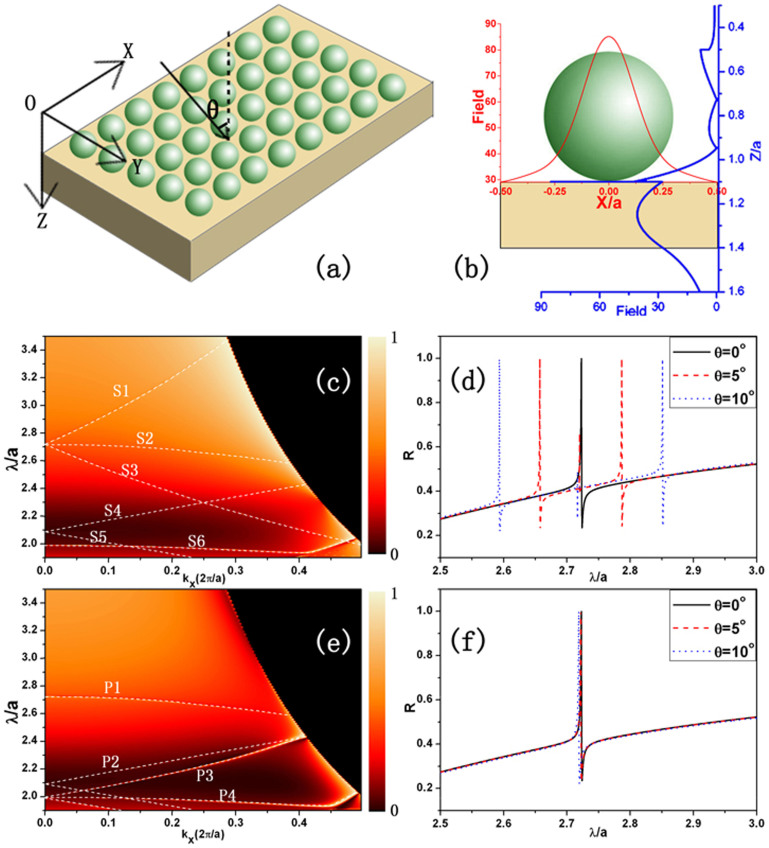
(a) Diagram of the sphere-slab structure and coordinate. The spheres are arranged in a square lattice with the lattice constant *a*. The radii of spheres are 0.3*a*. The slab is placed next to the spheres and the thickness is 0.3*a*. (b) shows the absolute value of the electric field in one primitive cell at 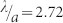. The incident wave is along Z-axis normally to the XY-plane, the amplitude of the incident field is 1 and the polarization is along X-axis. Red coordinate represents the field intensity distribution along the X-axis at the interface between the spheres and the slab (Y = 0, Z = 1.1); Blue coordinate corresponds to the field intensity distribution along the Z-axis at X = 0.01 and Y = 0.01. (c) and (e) describe the reflectivity R as a function of the reduced wavelength 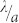 and the component of wave vector *k_x_* for S and P wave, respectively. Because the resonant peaks are too sharp to be displayed, we highlight the bound states with dashed lines. The boundary between the black and colored region is the light line. The corresponding reflectivity for the S and P waves at various incident angles are given in (d) and (f) as a function of the reduced wavelength 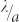.

We consider a double layer structure consisting of monolayer dielectric spheres with a square lattice and a dielectric slab as shown in Fig. 1(a). The distance between two neighbor spheres is taken as *a*, the relative permittivity and relative permeability of spheres are marked by *ε_s_* and *μ_s_*, respectively. The relative permittivity and permeability of the dielectric slab are expressed by *ε_d_* and *μ_d_*. For nonmagnetic materials, *μ_s_* = *μ_d_* = 1. By choosing appropriate parameters of the structure, we construct a BIC at the interface between the monolayer dielectric spheres and the dielectric slab, and realize to trap EM waves at such a position. Then, we put a sheet of monolayer graphene at such a position and explore the interaction between external EM fields and the graphene.

### Bound states in the continuum between monolayer dielectric spheres and a slab

Figure 1(c) and (e) show the reflectivity (R) of the double layer structure as a function of the reduced wavelength 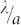 and the component of wave vector *k_x_* for the S wave and the P wave, respectively. Here the dielectric constants of spheres are taken as *ε_s_* = 4.0804, which correspond to *Si*_3_*N*_4_, and the dielectric constant of the slab is taken as *ε_d_* = 12.96 corresponding to *Si*. The radii of the spheres are *r* = 0.3*a* and the thickness of the slab is also taken as *D* = 0.3*a*. The results are obtained by the layer-multiple-scattering method^30^.

The sharp resonant features are clearly visible in Fig. 1(c) and (e) corresponding to the existence of trapped electromagnetic modes (quasistationary states) in the structure. For example, we observe a common resonant mode for both S and P waves at 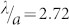 with the normal incident light. With the increase of *k_x_*, such a resonant mode for the S wave splits into three (S1, S2 and S3), the resonant mode for the P wave does not split (P1). Comparing them, we find that the modes S2 and P1 are overlap in all-angle range, they changes small with the increase of *k_x_*_._ This means that the change of such a resonant mode is also small with the change of the incident angle. This can be seen more clearly from Fig. 1(d) and (f).

Figure 1(d) and (f) show the corresponding reflectivity for the S wave and the P wave as a function of the reduced wavelength 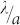 at various incident angles, respectively. The solid line, dashed line and dotted line correspond to *θ* = 0°, 5°, and 10°, respectively. For the S wave, the resonant peak showed a clear separation with the increase of the incident angle. In contrast, the positions of resonant peaks for the P wave keep unchanged with the change of the incident angle.

The phenomenon originated from the formation of BICs in the structure. In order to disclose it, in Fig. 1(b) we plot the electric field intensity at 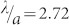 in one primitive cell along the X-axis (red coordinate) and Z-axis (blue coordinate) at the interface between the sphere and the slab. Here the incident wave is along the Z-axis, which is normally to the XY-plane. It is shown clearly that the EM field is mainly localized at the interface between the spheres and the slab, where the BICs appear. These BICs are generally understood as the result of the interference mechanism as described in Refs. 21–29, which depend on the structure and *k_x_* for a polarized wave at some certain frequency. This is the reason why we can observe the above phenomenon of mode splitting. Furthermore, this also imply that we can design BICs with proper choice of structure parameters^31^.

### Ultrasensitive optical absorption in grapheme

**Figure 2:**
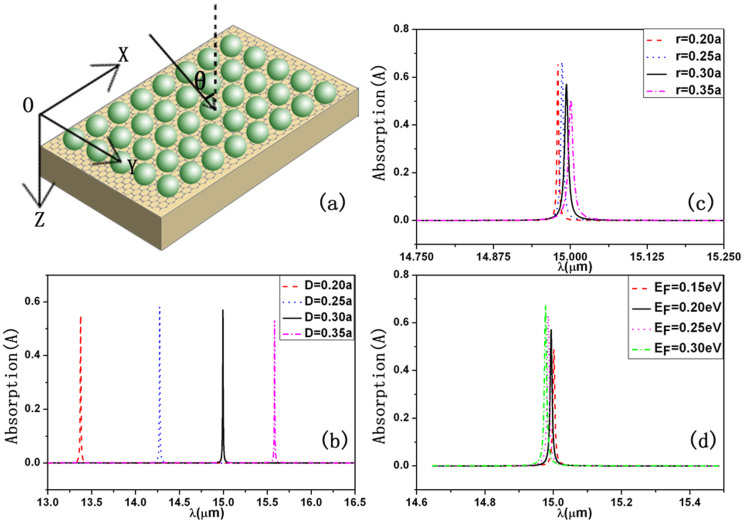
(a) Schematic diagram of the sphere-graphene-slab structure. (b), (c) and (d) show the absorption as a function of wavelength *λ* under the normal incident wave. Here *a* is taken as 5.5185 *μm*. (b) Various thickness of the slab at *r* = 0.3*a*. (c) Various sizes of the sphere at *D* = 0.3*a*. (d) Different *E_F_* at *r* = 0.3*a* and *D* = 0.3*a*. The other parameters are identical with those in Fig. 1.

In the following, we study the absorption properties of monolayer graphene when it is put at the interface between the monolayer dielectric spheres and the dielectric slab as shown in Fig. 2(a). In order to study the absorption properties of monolayer graphene in such a structure, we have developed layer-multiple-scattering method to include the graphene layer. Details of our calculated method are provided in the Methods section. Based on such a method, we have calculated absorption (A) with different tunable variables of the system. The absorption is defined from the requirement of energy conservation A = 1-T-R. Here T and R represent the transmittance and reflectivity, which are defined as the ratio of the transmitted, respectively the reflected, energy flux to the energy flux associated with the incident wave. In our calculations, the lattice constant is taken as *a* = 5.5185 *μm*, the other parameters for spheres and the slab are taken identical with those in Fig. 1. At room temperature 300 K and for mid-infrared wavelengths, the conductivity of monolayer graphene may be approximated with a Drude-like expression^32^,^33^,^34^,^35^,^36^

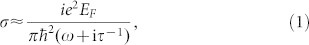
where *ω* is the angular frequency, *E_F_* represents Fermi level of graphene and the electron relaxation time *τ* is taken to be 0.3 ps based on typical values of the carrier mobility^37^.

Figure 2(b), (c) and (d) displays the calculated results of absorption as a function of wavelength for different tunable variables of the system. As we can see, a sharp strong resonant absorption peak always appears at some certain parameters. For example, the absorption of the resonant absorption peak, which appears at *λ* = 15 *μm* as shown in Fig. 2(b), can be close to 60%. The Q factor of such a resonant peak is 2156.3. Here the Q factor is defined by 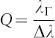, where *λ*_Γ_ is the center wavelength of the peak and Δ*λ* represents full width at half maximum of the peak. This means that the absorption peak is very sensitive to the wavelength. Furthermore, the resonant absorption peak strongly depends on the thickness of the slab as shown in Fig. 2(b), significant change of the peak magnitude can happen by tuning size of spheres when the thicknesses of the double layer are fixed as shown in Fig. 2(c), the absorption peaks are also tunable by the external electric field due to the electric field dependence of the Fermi surface of graphene as shown in Fig. 2(d). These phenomena are very benefit for biosensing, perfect filters and waveguides.

**Figure 3:**
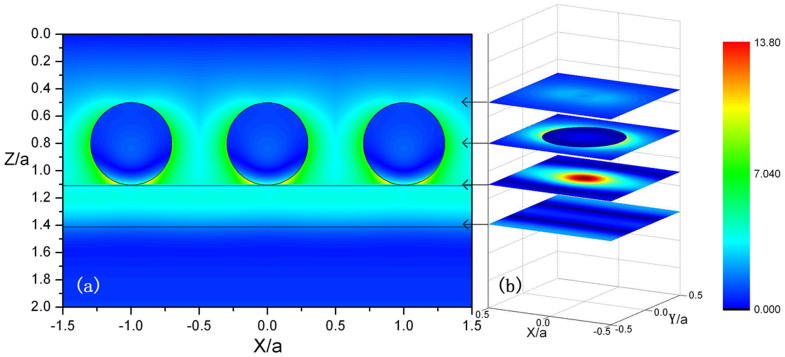
Distributions of the electric field intensity in the sphere-graphene-slab structure at the resonant absorption case. Here *λ* = 15 *μm*, *a* = 5.5185 *μm*, *r* = 0.3*a* and *D* = 0.3*a*. The other parameters are identical with those in Fig. 1. (a) Distributions of the electric field intensity in the XZ-plane at Y = 0. (b) Distributions of the electric field intensity in the XY-plane for one primitive cell with Z = 0.5, Z = 0.8, Z = 1.1 and Z = 1.39.

In order to disclose physical origin of the above phenomenon, in Fig. 3 we plot the distributions of the electric field intensity in the structure at the resonant absorption, which correspond to the resonant absorption peak at *λ* = 15 *μm* in Fig. 2(b). Figure 3(a) and (b) show the electric field intensity patterns in XZ-plane at Y = 0 and XY-plane for one primitive cell with various Z, respectively. The distributions of the electric field in the structure is very similar to that shown in Fig. 1(b), although the monolayer graphene has been put in the double-layer structure. We can see the maximum of field intensity appears in the graphene layer by the formation of BICs, which leads to strong interaction between the EM field and the graphene. This is the reason why there is such a strong absorption for the monolayer graphene in the present structure.

**Figure 4:**
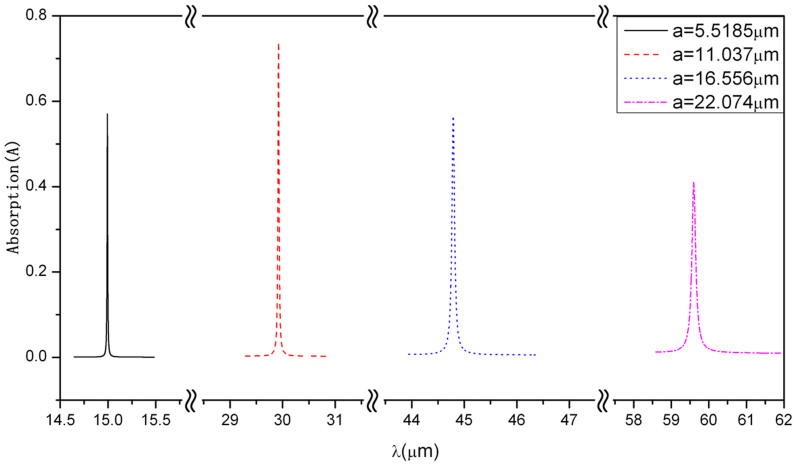
Absorption for the sphere-graphene-slab structure as a function of wavelength *λ* at various lattice constants under the normal incident wave. The other parameters are identical with those in Fig. 1.

Another advantage of such a design is that the resonant absorption peak can appear in the broad frequency range by choosing parameters of the structure. This is because the appearance of BICs strongly depends on the structure parameters. Through choosing suitable structure parameters, we can construct BICs and realize resonant absorptions of EM wave at any wavelength from mid-infrared to far-infrared band. For example, the resonant absorption peak appears at *λ* = 15.0 *μm* when the lattice constant is taken as *a* = 5.5185 *μm* as shown by the solid line in Fig. 4. It appears at *λ* = 29.9 *μm*, 44.8 *μm* and 59.6 *μm* (see dashed line, dotted line and dash-dotted line), respectively, when the lattice constant becomes 11.037 *μm*, 16.556 *μm* and 22.074 *μm*.

However, the feature of absorption peaks also exhibits some differences with the change of wavelength because the conductivity in graphene is a function of wavelength. With the increase of lattice constant, the shape of the absorption peak can be broaden and the Q factor will decrease. Thus, broad absorption peaks with low Q factor can also be obtained by designing. For example, as *a* = 22.074 *μm*, the Q factor of the absorption peak becomes 469.6. In such a case, the absorption has been below 40%. The sharp absorption peak with high Q factor has contributed to the ultrasensitive absorption devices. It is also beneficial to designing absorption devices with high error-tolerant rate by using absorption peaks with low Q factor.

**Figure 5:**
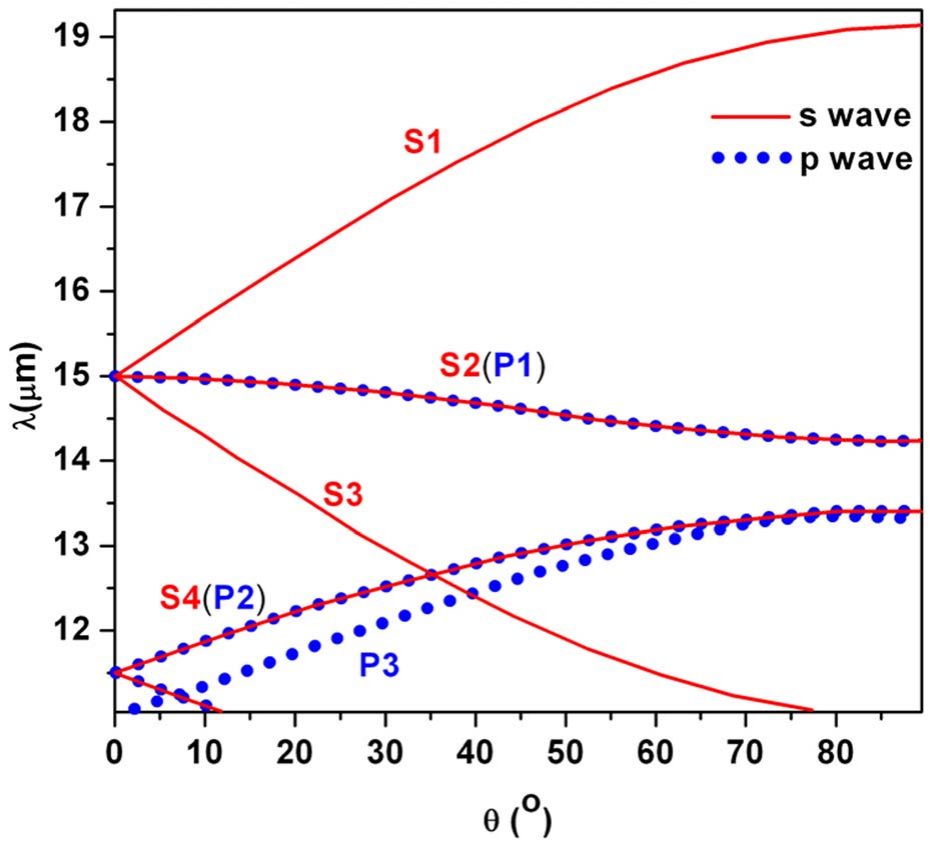
The absorption peaks as a function of the wavelength *λ* and the incident angle *θ*. Here *a* = 5.5185 *μm*. The solid lines and circle dotted lines represent absorption peaks for the S wave and the P wave, respectively. The other parameters are identical with those in Fig. 1.

The above discussions only focus on the case of wave with the normal incidence. In fact, the resonant absorption depends on the incident angle of the wave. This is because the BICs are related to the incident angle of the wave as shown in Fig. 1. The angle properties of absorption peaks depend on the choice of BIC modes, which can be seen clearly in Fig. 5. The solid and dotted lines represent absorption modes for the S wave and the P wave, respectively. Figure 5 shows the positions of absorption peaks as a function of the wavelength *λ* and the incident angle *θ*. It is seen clearly that the corresponding between the curves in Fig. 5 with those in Fig. 1(c) and (e) is very well.

Through the choice of BIC modes, the different situation of selective absorption can be realized. For example, curves S2(P1) and S4(P2) in Fig. 5, corresponding to curves S2 and S4 in Fig. 1(a) and P1 and P2 in Fig. 1(b), represent all-angle resonant absorptions for the S wave and the P wave at the same time. In contrast to the curve S4(P2), the curve S2(P1) is not very sensitive to the wavelength. When the wavelength is taken between 15.25 *μm* and 19.25 *μm*, the angle dependent resonant absorption peaks only exist for the S wave (curve S1 in Fig. 5), it is transparency for the P wave. Similarly, it is transparency for the S wave, the angle dependent resonant absorption peak only for the P wave (except a special point) is still exist as shown by curve P3. That is to say, we can realize different cases of selective absorption for the S wave and the P wave by designing the structure.

## Conclusions

Based on the BICs, we have designed sphere-graphene-slab structures to improve the interaction between the EM wave and the graphene, and realize strong absorption of EM field in the monolayer graphene. Such an absorption exhibits the following features:

1. The absorption peak can become very sharp, its Q factor is more than 2000, thus, it is ultrasensitive to the wavelength.
2. By taking suitable structure parameters, ultrasensitive strong absorption peak can appear at any wavelength from mid-infrared to far-infrared band. In the condition of certain structure parameters, the position of absorption peak can be also tunable by the external EM field.
3. Taking suitable BICs, not only we can realize all-angle absorption for the S wave and the P wave at the same time, but also can realize the selective absorption for the S wave and the P wave, respectively.

In the above discussions, only square lattice are taken for the monolayer spheres, in fact, for the other periodic structures such as triangular lattice, similar phenomena can be found. The calculated results and discussion will be given in the Supplementary Information.

## Methods

In this section, we provide the calculated method. We consider a sheet of monolayer graphene with conductivity *σ* is sandwiched between a homogeneous nonmagnetic medium with *ε*_1_ on the left-side and a homogeneous nonmagnetic medium with *ε*_2_ on the right-side. At the boundary we have
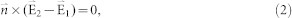

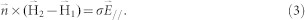


Here 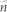 is the direction of the normal unit vector, 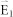 and 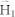 represent the EM fields in the left medium, 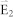 and 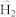 correspond to the fields in the right medium, 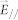 is the electric field component parallel to the interface of two mediums. As the single-layer graphene is quite thin, the effect of graphene on the scattering properties of the object has been attributed to the interfacial effect^38^,^39^,^40^,^41^.

With the incident wave from the left-side, the transmission and reflection coefficients at the interface can be expressed as 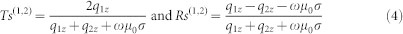
for the S wave, 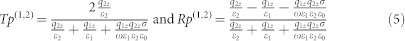
for the P wave. Here *μ*_0_ and *ε*_0_ are permittivity and permeability in vacuum and *q_jz_* is the normal component of the wave vector *q_j_*.

Based on the above analysis, we extend the layer-multiple-scattering method^30^ to include graphene layer. This method can calculate the transmittance, reflectivity and absorption of light by a slab of an infinite photonic crystal, which consists of a stack of identical slices parallel to a given surface. The electric fields outside each slice can be treated as 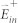, 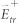 and 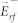, which represent the incident wave along + *z* direction and the corresponding transmitted wave and reflected wave, and 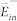, 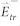, and 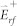 represent the corresponding waves along − *z* direction. These waves can be expanded as a set of plane waves
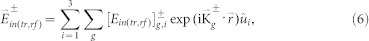
where 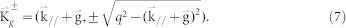
Here *q* is the wave vector, 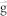 is the reciprocal lattice vector corresponding to the given primitive lattice, 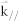 is the reduced wave vector which lies in the surface Brillouin zone. Outside the graphene, the relationships between the incident and the transmitted (reflected) waves are
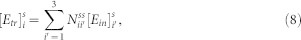

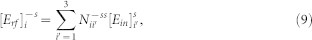
where the *s* = +(−) denotes a wave propagating to the right (left) and *i* = *x*, *y*, *z*. For given *ω* and *q*, the matrices *N^ss^*^′^ have the form
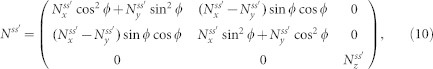
where *ϕ* is the azimuthal angle of *q*_//_ with respect to the x-axis and *q*_//_ is the parallel component of *q*. The elements in matrices *N^ss^*^′^ are 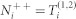, 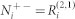, 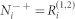 and 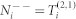, where 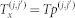, 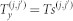, 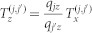, 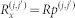, 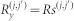 and 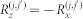.

We can further define the vectors 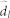 and 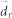 to construct four matrices *Q^η^*(*η* = I,II,III,IV) for this monolayer graphene and the plane waves outside the graphene as
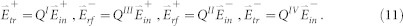
More details about the *Q^η^* matrices and the successiveness of different slices can be found in Ref. 30. In this way, we can calculate the absorption of the monolayer graphene using layer-multiple-scattering method mentioned above.

## Author Contributions

Numerical results and theoretical method are presented by M.Z., the idea and physical analysis are given by X.Z. All authors reviewed the manuscript.

## Supplementary Material

Supplementary Information Supplementary Information
