# Supplementary material for: Ultrasensitive optical absorption in graphene based on bound states in the continuum
Source: Sci Rep. 2015 Feb 5;5:8266. doi: 10.1038/srep08266 (PMC4317701; doi:10.1038/srep08266)
Supplement: Supplementary Information [file srep08266-s1.pdf]

Supplementary Information

Ultrasensitive optical absorption in graphene based on bound states in the continuum

Mingda Zhang,[1] and Xiangdong Zhang[2]

[1]Department of Physics, Beijing Normal University, Beijing 100875, China, [2]School of Physics, Beijing Institute of Technology, 100081, Beijing, China.

In the following, we present the calculated results for the double layer structure consisting of monolayer dielectric spheres with a triangular lattice and a dielectric slab as shown in Fig. S1(a). Figure S1(b) and (c) show the reflectivity (R) of the double layer structure as a function of the reduced wavelength λ/a and the component of wave vector k_x for the S wave and the P wave, respectively. The parameters of spheres and the slab are taken identical with those for the square lattice as shown in Fig. 1(a). The BICs for S and P waves are also found, which are similar to those in Fig. 1(c) and (e). For example, a common resonant mode for S and P waves appears at $\lambda/a = 2.45$ with the normal incidence. The difference is that the resonant mode for the P wave in the present structure also splits into three (P1, P2 and P3).

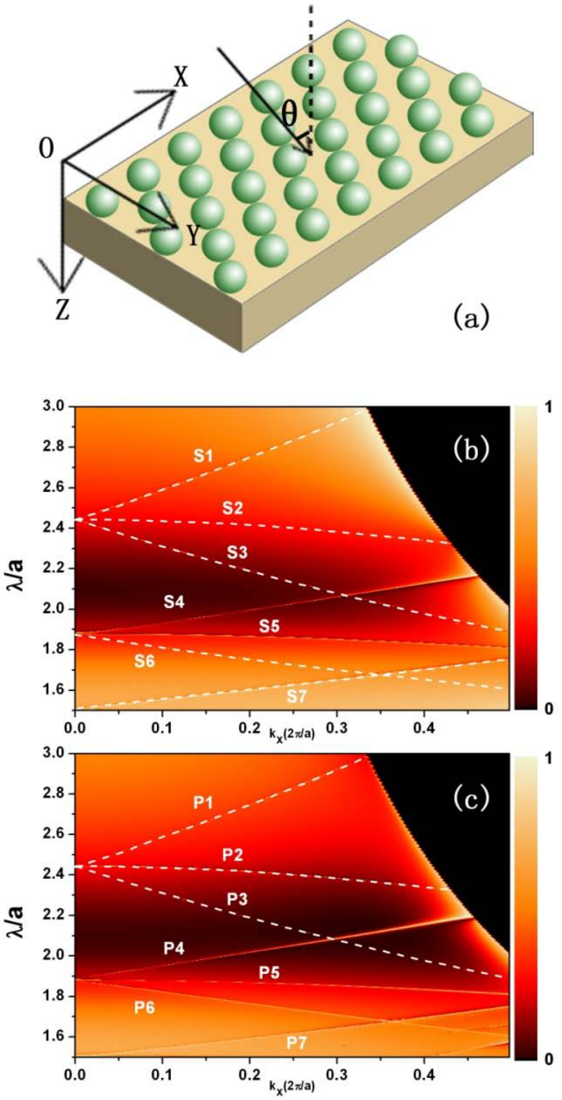

Figure S1 (a) Diagram of the sphere-slab structure and coordinate. The spheres are arranged in a triangular lattice with the lattice constant a. The radii of spheres are $0.3a$. The slab is placed next to the spheres and the thickness is $0.3a$. (b) and (c) describe the reflectivity R as a function of the reduced wavelength λ/a and the component of wave vector k_x for S and P wave, respectively. We highlight some bound states which are too sharp to be displayed with dashed lines. The boundary between the black and colored region is the light line.

If we put the monolayer graphene at the interface between the monolayer dielectric spheres and the dielectric slab as shown in Fig. S2(a), ultrasensitive strong optical absorption can also be realized. Figure S2(b) and (c) show the calculated results of absorption as a function of wavelength for different tunable variables of the system at the normal incident situation. It is shown clearly that sharp strong resonant absorption peaks appear at some certain parameters, which correspond to the BICs as shown in Fig. S1. Figure S2(b) describes the absorption as a function of wavelength for various thickness of the slab at $r = 0.3a$, and Figure S2(c) corresponds to the case for various sizes of the sphere at $D = 0.3a$. The absorption peaks as a function of the wavelength λ and the incident angle θ are plotted in Fig. S2(d). Here the lattice constant is also taken as $5.5185\mu m$. Ultrasensitive optical absorption in graphene, which is similar to the cases as shown in Fig. 2 and 5, has also been observed. However, we find that the features of absorption peaks for S and P waves exhibit very little difference because of high symmetry for the triangular lattice. For example, Three modes for the S wave (S1, S2 and S3) and the P wave (P1, P2 and P3) are common modes in all-angle range. The other three modes for the S wave (S4, S5 and S6) and the P wave (P4, P5 and P6) also display a few difference with the change of the incident angle.

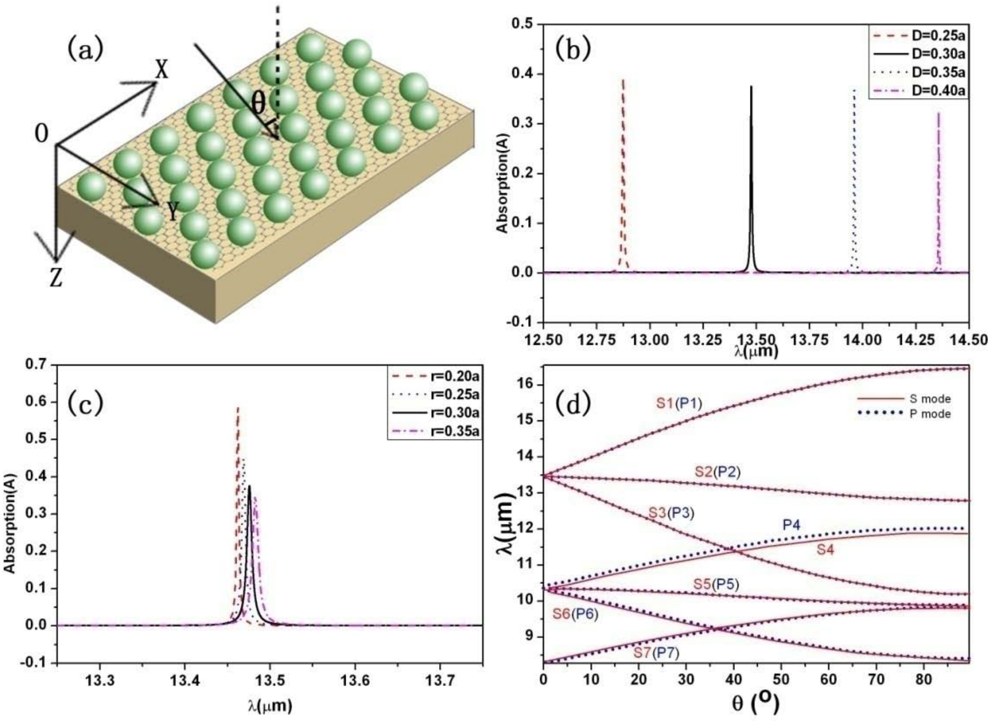

Figure S2 (a) Schematic diagram of the sphere-graphene-slab structure. (b) and (c) show the absorption as a function of wavelength λ under the normal incident wave. (b) Various thickness of the slab at $r = 0.3a$. (c) Various sizes of the sphere at $D = 0.3a$. (d) shows the absorption peaks as a function of the wavelength λ and the incident angle θ. Here $a = 5.5185\mu m$.
